# Universal HIV testing and the impact of late diagnosis on disease stage among adults in urban Ethiopia

**DOI:** 10.1186/s41182-023-00494-z

**Published:** 2023-01-18

**Authors:** Yimam Getaneh, Jemal Ayalew, Qianxin He, Adamu Tayachew, Abdur Rashid, Desta Kassa, Sileshi Leulseged, Lingjie Liao, Feng Yi, Yiming Shao

**Affiliations:** 1 State Key Laboratory for Diagnosis and Treatment of Infectious Diseases, National Clinical Research Center for Infectious Diseases, Collaborative Innovation Center for Diagnosis and Treatment of Infectious Diseases, The First Affiliated Hospital, College of Medicine, Zhejiang University, Hangzhou, China; 2 Ethiopian Public Health Institute, Addis Ababa, Ethiopia; 3 School of Medicine, Nankai University, Tianjin, China; 4 College of Health Science, School of Medicine, Addis Ababa University, Addis Ababa, Ethiopia; 5 State Key Laboratory for Infectious Disease Prevention and Control, National Center for AIDS/STD Control and Prevention, Chinese Center for Disease Control and Prevention, Beijing, 102206 China

**Keywords:** Disease stage, HIV/AIDS, Late diagnosis, Universal testing

## Abstract

**Background:**

Treatment as prevention evolved into the universal HIV test-and-treat (UTT) strategy, which entails testing to the general population and treatment to every people living with HIV. We investigated universal testing (UT) performance and its determinants in urban Ethiopia and explore magnitude of late diagnosis and its impact on disease stages.

**Method:**

We used data from the Ethiopia Population Based HIV Impact assessment (EPHIA), conducted in 2017/2018 which was a cross-sectional and household-based study. For current analysis, we considered self-report first diagnosis to estimate universal testing irrespective of their serostatus and also consider HIV LAg avidity vs viral load vs plasma antiretroviral drug level algorithm to categorize the late diagnosis. We finally evaluate disease stages using CD4 count and viral load. A 2-level multilevel mixed-effect logistic regression model was employed. The effects of individual-level predictors were quantified by the estimates from the fixed-effect part of the model with *p*-value < 0.05.

**Result:**

Data were collected from 18,926 adults among those 29.4% of people living in Urban Ethiopia were never tested for HIV. Never tested females was 26.4% (95% CI = 25.3; 27.5). Never tested among divorced and widowed were 19.4% (95% CI: 17.3; 21.8) and 28.3% (95% CI: 24.6; 32.2), respectively. Never tested among elderly and youth were high (28.3% among 45–54 years old) to (41.2% among 55–64 years old) to 47.8% among 15–24 years old. Overall, late HIV diagnosis among adults in urban Ethiopia was 25.9% (95% CI: 21.7, 30.2). Late diagnosis varies by region ranged from 38.1% in the Gambella to 5.8% in Benishangul Gumuz. Advanced immune suppression (CD4 count < 350 cells/µl) among newly diagnosed long-term infection were significantly higher compared to those who were recently infected which accounted 47.8% (95%CI = 33.2–52.1) and 30.9% (95%CI = 21.3–32.2), respectively. Moreover, Viral load suppression were significantly lower among those who were late diagnosed 26.1% (95%CI = 13.6–33.8) compared to those of newly infected 89.6% (95%CI = 76.2; 93.4).

**Conclusion:**

With the aim of UT for high risk and priority population, the low rate of HIV testing among widowed, elderly, young adolescent and women in urban Ethiopia calls for enhanced HIV testing. Moreover, the low HIV testing and high late diagnosis among the high-burden regions calls for region-specific intervention. Advanced disease stages as a result of the high proportion of late diagnosis may impact on fueling community transmission and hinder treatment outcome among PLHIV.

## Background

Human immunodeficiency virus (HIV) remains one of the leading causes of morbidity and mortality in sub-Saharan Africa. Although global commitment to control the HIV/AIDS pandemic has increased significantly in recent years, the virus continues to spread with alarming and increasing speed [1]. Globally, HIV Testing Service (HTS) and antiretroviral therapy (ART) have been scaled up substantially. In 2005 it was estimated that in Africa only 10% of people with HIV were aware of their HIV status and that, globally, only 12% of people who wanted to test for HIV were able to do so. Nearly 15 years later it is now estimated that 85% of all people with HIV in eastern and southern Africa, and nearly 80% of people with HIV worldwide, know their status. With the offer of immediate ART initiation and improved treatment options, access to and uptake of treatment has increased. Now, most people with HIV who know their status are accessing treatment and care [2].

In Ethiopia, the burden of HIV is high as in elsewhere in sub-Saharan Africa (SSA), accounting for about 720,000 people living with HIV (PLHIV) and 27,104 newly diagnosed cases by 2020. Overall prevalence of HIV in Ethiopia was therefore, 0.96% while the prevalence in urban was 3% by 2020 [3].

Targeted HIV testing among the key and priority population and early HIV diagnosis is one of the effective ways to prevent the spread of the epidemic [4]. This helps to lower the viral load (VL) and improve CD4 count, resulting in a dramatic reduction of the risk of morbidity and mortality among PLHIV, and in decreasing HIV transmission by greater than 90% [5, 6]. The World Health Organization (WHO) recommend “universal test and treat” (UTT) approach to end AIDS by 2030. UTT is approach which recommends all population at risk to be screened for HIV infection and those diagnosed HIV positive receive immediate treatment regardless of CD4 count [7]. In Ethiopia at risk and priority populations to be covered under UTT strategy were women, people living in hotspot areas (Gambella and Addis Ababa), female sex workers, widowed and separated, elderly and families of PLHIV [8]. Understanding status of universal testing among at risk population and assessing determinants of late diagnosis and its impact on disease stage is of a priority for the program to focus areas of intervention.

Late diagnosis is associated with: increased HIV-related morbidity and mortality, shorter survival, poor response to treatment, increased healthcare costs and increased rates of HIV transmission. If a person is diagnosed and treated for HIV early in the course of infection before severe impairment of the immune system has occurred, life-expectancy may approach that of the general population [9–13].

In Ethiopia, evidences on the universal testing in urban Ethiopia among the different subpopulation and magnitude and determinants of late diagnosis and its impact on the different disease stage are limited. Hence, we evaluated status of universal testing and late diagnosis among at risk and priority population in urban Ethiopia and assess the impact of late diagnosis on disease stages.

## Methods

### Study design and setting

This analysis involved the Ethiopian Population Based HIV Impact Assessment (EPHIA) data, which was collected through urban-representative, cross-sectional and household-based survey conducted in urban Ethiopia from October 2017 to April 2018.

The domain of analysis for the universal testing was all adult people (age > 15 years old) in urban Ethiopia (which was further disaggregated to evaluate testing among the different key and priority population in Ethiopia) while for the late diagnosis, we considered only adult PLHIV. For the categories of the different disease stages we considered CD4 count < 350 cells/µl or Viral load ≥ 1000 copies/ml as defined by the world Health organization [14, 15].

Hence, for the universal testing we included all adults stayed overnight in the household (de facto). Moreover, for the late diagnosis and classification of disease stages, we considered only adult people living with HIV (tested positive). HIV-negative population was excluded from analysis of late diagnosis and disease stages.

### Study population and sampling procedures

EPHIA used a two-stage cluster sampling design with stratification into small and large urban areas. In the first stage 393 enumeration area (EAs)/clusters were selected using a probability proportional to size method based on EAs created by CSA for the 2007 Ethiopia Population and Housing Census, which included 17,339 EAs containing around three million households. The 393 EAs were further stratified by nine regional states and two city administrations: Tigray, Afar, Amhara, Oromia, Somali, Benishangul Gumuz, SNNPR, Gambella, Harari, Addis Ababa, and Dire Dawa. In second stage sampling, 30 households were randomly selected from each EA using an equal probability method, resulting in a total number of 11,810 households where 19,136 adults reside in.

### Data collection

Survey Staff Fieldwork started at the beginning of October 2017 and was completed in April 2018. Fieldwork was conducted by 31 locally hired field teams composed of a team leader, six nurse interviewers and two drivers, who were locally hired. Field teams included both male and female staff members who spoke the languages used in the areas to which they were deployed. A total of about 328 field staff (six field coordinators, 31 team leaders, 211 nurse interviewers [93 testers and 118 interviewers], six community-mobilization coordinators, and 74 drivers) participated in data collection. The field teams were supervised by 31 team leaders, six field coordinators and managed by central staff, who guided and oversaw data collection activities, performed quality checks, and provided technical support. In addition, the laboratory staff was organized at different levels (central laboratory staff, regional field supervisors, onsite laboratory supervisors, satellite lab technicians, and satellite lab logisticians). Laboratory staff were trained in specimen management, including sample processing, labeling, and quality assurance (QA). Central laboratory staff were trained in VL measurement, early infant diagnosis, HIV confirmatory testing, and testing for recent HIV infection using the limiting antigen (LAg) avidity enzyme immunoassay (EIA). National and international monitors periodically conducted direct observation of data collection activities in the field and in the laboratories to provide technical support and ensure quality.

### Laboratory-based biomarker testing to identify late diagnosis

HIV-1 VL (HIV RNA copies per mL) of confirmed HIV-positive participants was measured from plasma using the Roche (COBAS® AmpliPrep/COBAS® TaqMan® HIV-1 Test, Roche Diagnostics, Indianapolis, Indiana, United States) and from DBS using Abbott m2000 System (Abbott Molecular Inc., Chicago, Illinois, United States). Both instruments consist of two separate instruments, the sample preparation (Ampli prepap and m2000sp, which carries out automated extraction, purification, and preparation of HIV-1 RNA), and the Cobas Taqman-96 and m2000rt (which amplifies, detects, and measures the HIV-1 RNA load). In Cobas Taqman-96, 1 mL of plasma protocol was used, while the open-mode protocol for the Abbott Real Time HIV-1 assay was used to measure VL from DBS samples from adults with insufficient volume of plasma.

**Fig. 1:**
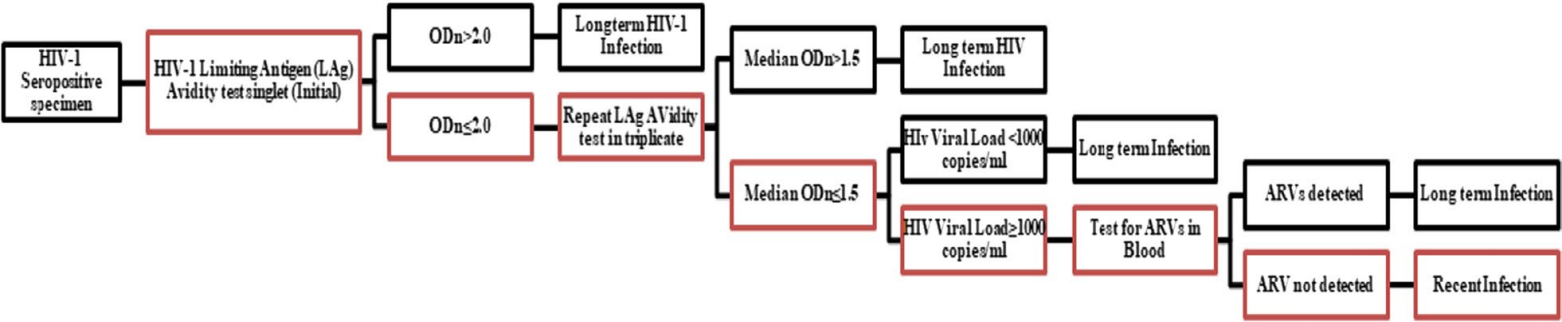
HIV-1 recent infection testing algorithm (LAg/VL/ARV algorithm)

To distinguish recent from long-term HIV infections, in order to estimate incidence, the study used two different laboratory-based testing algorithms. Each algorithm employed a combination of assays: 1) HIV-1 LAg-Avidity EIA (Sedia Biosciences Corporation, Portland, Oregon, United States) and VL and 2) HIV-1 LAg Avidity EIA, VL, and ARV detection (Fig. 1).

Specimens with median normalized optical density (OD), ≤ 1.5 using LAg avidity testing was classified as potential recent infections, and their VL results were assessed. Specimens with VL < 1000 copies/mL were classified as long-term infections, while those with VL ≥ 1,000 copies/mL were classified as recent infections. In the ARV-adjusted algorithm, specimens with VL ≥ 1000 copies/ml and with detectable ARVs were classified as long-term infections. Specimens with VL ≥ 1000 copies/mL and without detectable ARVs were classified as recent infections.

Qualitative screening, for detectable concentrations of ARVs, was conducted on DBS specimens from all HIV-positive adults by means of high-resolution liquid chromatography coupled with tandem mass spectrometry. The method used for ARV detection was a modified version of the methodology described by Koal et al. This qualitative assay was highly specific, as it separates the parent compound from the fragments, and highly sensitive, with a limit of detection of 0.02 μg/mL for each drug and a signal-to-noise ratio of at least 5:1 for all drugs. As detection of all ARVs in use at the time of the study was cost-prohibitive, three ARVs, efavirenz, lopinavir, and nevirapine, selected as markers for the most commonly prescribed first and second line regimens. Samples from participants who had suppressed viral loads and/or reported being on ART, but had no evidence of the first three compounds, were tested for nevirapine. These ARVs were also selected based on their relatively long half-lives, allowing for a longer period of detection following intake. ARV detection was performed by the Division of Clinical Pharmacology of the Department of Medicine at the University of Cape Town in South Africa.

### Statistical analysis

For this analysis, demographic, behavioral and environmental factors were considered as independent variable. HIV testing, late diagnosis and disease stages were also considered as dependent variables.

A descriptive analysis was done to assess demographic and individual characteristics of the study population. Bivariate analysis with cross-tabulation and 95% confidence interval (CI) were employed to examine the association between the outcome variable (HIV testing and late diagnosis) and the selected predictor variables.

A multilevel logistic regression model was fitted to assess for regional variation in the two dependent variables and identify their association with the independent variables considered in the study. A 2-level multilevel mixed-effect logistic regression model was employed. All the independent variables categorized as individual-level variables were considered level-1 variables and the region as level-2 variables. In the multilevel regression model, the effect of level-2 variable (regional) was quantified by intra-class correlation (ICC), the proportion of total variation in the response variable accounted for by the between-regional variation. The effects of individual-level predictors were quantified by the estimates from the fixed-effect part of the model with a p-value less than 0.05 or 95% CI.

The significance in improvement of multilevel model over the usual standard logistic model was checked by the Chi-square test. A significance Chi-square test result indicated that the multilevel model better fitted over the standard logistic model. Data analysis was done using Stata Statistical Software v16.0 (TX: Stata Corp LLC).

## Results

### Demographic characteristics of the study participants

Overall, data were collected from 18,926 adults. The higher proportion of population 4708 (33.5%) were from Oromoia region while 3280 (18.8%) and 2795 (16.1%) were from Amhara and SNNPR regions. With regard to the marital status, 8896 (46.5%), 7529 (35.9%) and 1829 (8.6%) were married, never married and divorced, respectively. While study participants disaggregated educational status, 7098 (35%), 5783 (28.8%) and 4682 (24.3%) attended primary, secondary and more than secondary education, respectively. Majority 18,176 (90.1%) were in the age from 15 to 49 years old while 1994 (9.9%) were in the age range from 50 to 64 years old (Table 1).

**Table 1:** Demographic characteristics of the adult population in Urban Ethiopia (2017/18)

	Male		Female		Total	
Characteristic	Percent	Number	Percent	Number	Percent	Number
Region						
Tigray	6.2	467	8.3	935	7.2	1402
Afar	1.3	355	1.3	497	1.3	852
Amhara	19.3	1334	18.4	1946	18.8	3280
Oromia	33.6	1871	33.5	2837	33.5	4708
Somali	1.2	367	1.3	590	1.3	957
Benishangul Gumuz	1.4	355	1.2	468	1.3	823
SNNPR	18.1	1289	14.1	1506	16.1	2795
Gambella	0.7	381	0.5	443	0.6	824
Harari	0.6	286	0.7	448	0.7	734
Addis Ababa	16.6	1030	19.4	1948	18	2978
Dire Dawa	1.1	277	1.4	540	1.2	817
Marital status						
Never married	42.3	3552	29.5	3977	35.9	7529
Married	47.2	3585	45.8	5311	46.5	8896
Living together	5.7	416	4.9	579	5.3	995
Divorced or separated	4.2	356	12.9	1473	8.6	1829
Widowed	0.6	57	6.9	737	3.8	794
Education						
No education	5.7	470	18.2	2068	11.9	2538
Primary	33	2634	37	4464	35	7098
Secondary	31.3	2536	26.3	3247	28.8	5783
More than secondary	30	2349	18.5	2333	24.3	4682
Wealth quintile						
Lowest	17.1	1460	16	2057	16.5	3517
Second	19.1	1567	16.4	2059	17.7	3626
Middle	20.3	1648	19.1	2358	19.7	4006
Fourth	21.7	1698	22.1	2653	21.9	4351
Highest	21.9	1639	26.5	3030	24.2	4669
Employment status						
Employed	58.7	4523	36.4	4155	47.5	8678
Not employed	41.3	3481	63.6	7984	52.5	11,465
Age						
15–24	34.7	2910	34.9	5004	34.8	7914
15–49	90.3	7146	89.9	11,030	90.1	18,176
50–64	9.7	866	10.1	1128	9.9	1994
15–64	100	8012	100	12,158	100	20,170

### HIV testing by different characteristics in urban Ethiopia

A total of 18,926 adults aged 15 to 64 years were included in the analysis to assess self-reported awareness of HIV testing in urban Ethiopia. Overall, 29.4% of them reported that they never got tested for HIV. The proportion never tested for HIV ranged from 18.8% in the Harari region to 20.3% in Afar to 32.6% in Oromia to 69.1% in the Somali region. Proportion never tested in small urban areas was 32.1%, and 26.8% among adults in large urban areas (Table 1).

As shown in Table 2, the proportion of never tested among adult males was significantly higher, 32.4% (95%: 31.0, 33.9) than females, 6.4% (95%: 25.3, 27.5). Nearly half of those who were never married, 49.5% (95% CI: 47.7, 51.3) were never tested. The proportion never tested was more than double among the married 17.3% (95% CI: 16.1, 18.6) and the divorced or separated 19.4% (95% CI: 17.3, 21.8), and was almost twice among widowed, 28.3% (95% CI: 24.6, 32.2).

**Table 2:** Prevalence of self-reported HIV testing history by demographic and behavioral characteristics in urban Ethiopia (2017/18)

			Never tested	
Variables	Ever tested	Never tested	%	95% CI
Region				
Tigray	1029	314	22.5	(19.6, 25.6)
Afar	641	161	20.3	(16.5, 24.8)
Amhara	2157	818	26.6	(23.8, 29.7)
Oromia	3006	1440	32.6	(30.2, 35.1)
Somali	274	629	69.1	(59.2, 77.5)
Benishangul Gumuz	567	227	28..6	(24.2, 33.4)
SNNPR	1794	864	32.4	(30, 35)
Gambella	565	192	25	(20, 30.9)
Harari	552	138	18.8	(14.1, 24.5)
Addis Ababa	2015	753	25.2	(23.1, 27.3)
Dire Dawa	565	204	25.8	(21.1, 31)
Area				
Small	6155	2988	32.1	(30.4, 33.8)
Large	7031	2752	26.8	(25, 28.6)
Gender				
Female	8250	3221	26.4	(25.3, 27.5)
Male	4936	2519	32.4	(31, 33.9)
Marital status				
Never married	3464	3546	49.5	(47.7, 51.3)
Married or living together	7748	1610	17.3	(16.1, 18.6)
Divorced or separated	1393	323	19.4	(17.3, 21.8)
Widowed	1393	226	28.3	(24.6, 32.2)
Educational status				
Not educated	1550	821	33.5	(31, 36.1)
Primary	4499	2223	32.3	(30.5, 34.1)
Secondary	3624	1805	32.4	(30.1, 34.3)
More than secondary	3464	882	19.8	(18.3, 21.4)
Employment in past 12 months				
Did not work	6724	4082	37	(35.6, 38.5)
Worked	6445	1655	21	(19.9, 22.1)
Age in years				
15–24	3997	3421	47.8	(46.1, 49.5)
25–34	4830	804	14.5	(13.2, 16)
35–44	2563	555	17	(15.2, 19)
45–54	1148	486	28.3	(25.6, 31.2)
55–64	648	474	41.2	(38.2, 44.2)
Wealth quintile				
Lowest	1985	1298	39.3	(36.5, 42.2)
Second	2329	1066	30	(27.9, 32.2)
Middle	2747	1037	26.3	(24.3, 28.4)
Fourth	3006	1097	26.6	(24.4, 28.9)
Highest	3119	1242	27.2	(25.5, 29)
Drank alcohol				
Not reported drinking	7704	4031	33.2	(31.9, 34.5)
Yes reported drinking	5481	1709	24.2	(22.7, 25.7)
Total	13,186	5740	29.4	(28.3, 30.5)

In terms of wealth quintile, 39.3% of the adults who were in the lowest quintile had never tested for HIV, significantly more than those who were in the second, third, fourth, and highest quintiles (30.0%, 26.3%, 26.6%, and 27.2%) respectively, with non-overlapping 95% CIs. Similarly, the proportion of never tested for HIV among adults who were not employed (during the last 12 months) preceding the study was 37.0%, significantly more than almost twice among employed (during last 12 months) (21.0%) with the non-overlapping 95% CIs. Among the behavioral characteristics, the proportion of never tested for HIV was significantly higher in adults who did not report drinking alcohol, 33.2% (95% CI: 31.9, 34.5) compared with adult who used alcohol, 24.2% (95% CI: 22.7, 25.7).

### Late HIV diagnosis

Among adults aged 15 to 64 years tested for HIV, 614 (3.0%) adults tested positive. A total of 611 PLHIV were included in the analysis to assess late HIV diagnosis. Overall, late HIV diagnosis among adults in urban Ethiopia was 25.9% (95% CI: 21.7, 30.2). Despite the diagnosis, 3.4% of PLHIV were also not linked to HAART. The proportion of adults who tested for HIV late ranged from 38.1% in Gambela to 34.7% in Addis Ababa to 5.8% in Benishangul Gumuz to none in the Somali region (Table 3).

**Table 3:** Prevalence of late HIV diagnostics among people living with HIV (PLHIV) in urban-based population of Ethiopia (2017/18)

			Newly diagnosed but long-term infection	
Variables	Recent infection + self-reported positive	Newly diagnosed but long-term infection	%	95% CI
Region				
Tigray	35	4	10.3	(4.2, 23.1)
Afar	24	8	24.9	(14.4, 39.6)
Amhara	99	19	18.1	(10.6, 29.2)
Oromia	108	41	31	(22.8, 40.5)
Somalia	8	0	0	(0, 0)
Benishangul Gumuz	19	1	5.8	(1, 26.7)
SNNPR	32	17	34.7	(20.7, 51.9)
Gambella	28	16	38.1	(17.8, 63.6)
Harari	22	10	28.8	(15.7, 46.7)
Addis Ababa	62	26	30.5	(21.8, 40.9)
Dire Dawa	29	6	18.5	(8.7, 35.2)
Gender				
Female	363	98	20.8	(17.3, 24.9)
Male	103	50	36.8	(28, 46.6)
Marital status				
Never married	49	22	30.3	(19.9, 43.3)
Married or living together	212	73	29.2	(22.3, 37.3)
Divorced or separated	114	30	21.5	(14.6, 30.6)
Widowed	90	22	20.6	(13.6, 28.7)
Educational status				
Not educated	98	23	16.4	(10.7, 24.4)
Primary	217	74	28.4	(22.4, 35.3)
Secondary	111	30	25.5	(18.2, 34.4)
More than secondary	38	20	35.4	(22.3, 51.1)
Age				
15–24	35	27	43.5	(28.6, 59.6)
25–34	134	41	26.6	(19.3, 35.4)
35–44	186	48	23.2	(16.7, 31.3)
45–54	79	25	25.2	(17, 35.5)
55–64	32	7	19.5	(9.5, 36)
Wealth quintile				
Lowest	77	26	31.7	(20.8, 45)
Second	85	23	17.8	(10.1, 29.5)
Middle	116	27	21.4	(14.6, 30.2)
Fourth	107	40	29	(20.4, 39.4)
Highest	81	32	30.5	(21.5, 41.3)
Drank alcohol				
Not reported drinking	319	78	19.7	(15.3, 25.1)
Yes reported drinking	147	70	36.1	(29.3, 43.5)

As shown in Table 4, the proportion of late diagnosis of HIV among adult males was significantly higher, 36.8% (95%: 28.0, 46.6), than females, 20.8% (95%: 17.3, 24.9). The proportion of never married, married or living together PLHIV was slightly higher among those with late diagnosis, 30.3% and 29.2%, respectively, compared to the divorced, separated and widowed, 21.5% and 20.6%, respectively.

**Table 4:** Adjusted odds ratio (AOR) and 95% confidence interval (CI) for AOR of the multilevel mixed-effect logistic regression for the associated factors for never tested HIV in urban Ethiopia (2017/18)

Variables	AOR	p-values	95% CI
Area			
Small	1.24	0.00	(1.14, 1.36)
Large	1		
Gender			
Female	1		
Male	1.39	0.00	(1.29, 1.5)
Marital status			
Never married	1	0.00	
Married or living together	0.18	0.00	(0.16, 0.2)
Divorced or separated	0.19	0.00	(0.17, 0.23)
Widowed	0.2	0.00	(0.16, 0.25)
Educational status			
Not educated	3.77	0.00	(3.27, 4.34)
Primary	2.34	0.00	(2.1, 2.6)
Secondary	1.62	0.00	(1.45, 1.79)
More than secondary	1		
Age in years			
15–24	2.49	0.00	(2.24, 2.77)
25–34	1	0.00	
35–44	1.54	0.00	(1.35, 1.75)
45–54	3.14	0.00	(2.72, 3.64)
55–64	5.26	0.00	(4.48, 6.17)
Wealth quintile			
Lowest	1	0.00	
Second	0.73	0.00	(0.65, 0.82)
Middle	0.67	0.00	(0.59, 0.75)
Fourth	0.68	0.00	(0.6, 0.77)
Highest	0.82	0.00	(0.72, 0.93)
Drank alcohol			
Not reported drinking	1.37	0.00	(1.26, 1.48)
Yes reported drinking	1	0.00	
Constant	0.34	0.00	(0.23, 0.5)
Random intercept (Region)			
Var(Uoi)	0.35		(0.15, 0.82)
Region ICC	0.096		(0.043, 0.199)

LR test vs. logistic model: Chi-2(01) = 547.62 Prob >  = Chi-2 = 0.0000

Over one-third (35.4%) of the adult PLHIV attended secondary education and above were diagnosed late, followed by those with primary education (28.4%), secondary education (25.5%), and those who never attended school (16.4%). The highest proportion of late HIV diagnosis was observed among the adult PLHIV was in younger age group, 15–24 years (43.5%), followed by those 25–34 years (26.6%), and those 45–54 years (25.2%). Late HIV diagnosis was the lowest among the 55–64 year age group (19.5%) (Table 4).

PLHIV among adults with the lowest, fourth, and the highest quintiles had a higher proportion of late HIV diagnosis, 31.7%, 30, 5%, and 29.0%, respectively, while lower proportion was seen in second (17.8%) followed by the middle quintile (21.4%). The proportion of late diagnosis among PLHIV who used alcohol was 36.1%, almost twice compared with adults not reported using alcohol (19.7%).

### Magnitude of never tested, burden of HIV and newly diagnosed long-term infections among people in urban Ethiopia, disaggregated by region

**Fig. 2:**
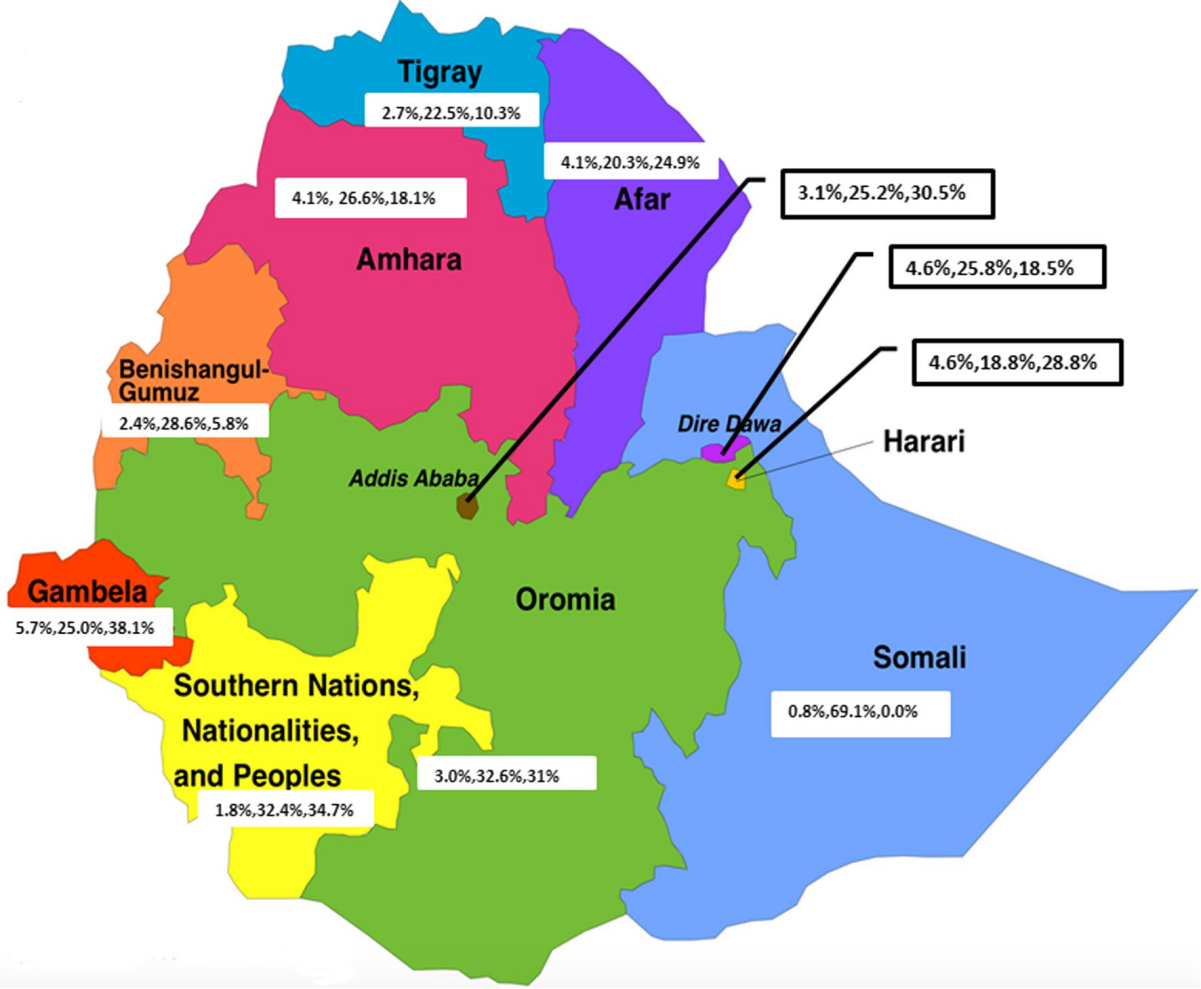
Prevalence of HIV, magnitude of never tested and newly diagnosed long-term infection, respectively, among people in urban Ethiopia disaggregated by region, 2017/18

As shown in Fig. 2 below, prevalence of HIV among the regional administrations in Ethiopia was heterogonous, ranging from 5.7% in Gambella to 0.8% in Somali. Never tested among people in urban Ethiopia also ranged from 69.1% in Somali to 20.3% in Afar regions. Newly diagnosed long-term infection was the highest in Gambella 38.1 to 0.0% in Somali region.

### Determinants for never tested for HIV

The results multilevel mixed-effect logistic regression model on diagnosis show that the model fitted well for the data over the standard logistic regression to assess determinants for never tested for HIV (*X*^2^ = 547.62, *p* < 0.01). The result of ICC of the two-level multilevel model revealed that about 9.6% of the variation in the likelihood of never tested for HIV was explained by the variation among the regions in urban Ethiopia (95% CI: 0.043, 0.199) (Table 4).

Adults aged 15–24 years (AOR: 2.49; 95% CI: 2.24, 2.77), 35–44 years (AOR: 1.54; 95% CI: 1.35, 1.75), 45–54 years (AOR: 3.14; 95% CI: 2.72, 3.64), and those 55–64 years (AOR: 5.26; 95% CI: 4.48, 6.17) were more likely to have never tested for HIV, respectively, as compared to those 25–34 years of age. The odds of being never tested for HIV among adults living in small urban areas were 1.24 times higher than those living in large urban areas (AOR: 1.24; 95% CI: 1.14, 1. 36). Being male was significantly and positively associated with never tested status compared with the female counterparts (AOR: 1.39; 95% CI: 1.29, 1.50) (Table 4).

Adults who were married or living together, divorced or separated, and widowed had 82.0% (AOR: 0.18; 95% CI: 0.16, 0.20), 81.0% (AOR: 0.19; 95% CI: 0.17, 0.23), and 80.0% (AOR: 0.20; 95% CI: 0.16, 0.25), respectively, lower odds of being never tested for HIV compared to their never married counterparts. In terms of education level of respondents who never attended school (AOR: 3.77; 95% CI: 3.27, 4.34), primary (AOR: 2.34; 95% CI: 2.10, 1.79), and secondary education (AOR: 1.62; 95% CI: 1.45, 1.79) were more likely to never tested for HIV, respectively as compared to those who had above secondary education (Table 4).

Adults who were in second, middle, fourth, and highest wealth quintiles had 27.0% (AOR: 0.73; 95% CI: 0.65, 0.82), 33.3% (AOR: 0.67; 95% CI: 0.59, 0.75), 32.0% (AOR: 0.68; 95% CI: 0.60, 0.77), and 18.0% (AOR: 0.82; 95% CI: 0.72, 0.93), respectively, lower odds of being never tested compared to their counterparts in the lowest quintiles. The odds of being never tested for HIV among adults aged 15 to 64 who had not drink alcohol (AOR 1.37; 95% CI; 1.26, 1.48) was 1.37 times higher compared to those who used alcohol.

### Determinants for late HIV diagnosis

The results on the diagnosis of multilevel mixed-effect logistic regression model showed that the model fitted well for the data over the standard logistic regression to assess determinants for late diagnosis for HIV (*X*^2^ = 6.6, *p* < 0.01). The result of ICC of the two-level multilevel model revealed that about 6% of the variation in the likelihood of never tested for HIV was explained by the variation among the regions in urban Ethiopia (95% CI: 0.012, 0.257) (Table 5).

**Table 5:** AOR and 95% CI for AOR of the multilevel mixed-effect logistic regression for the associated factors for lately tested HIV among PLHIV in urban of Ethiopia (2017/18)

Variables	AOR	p-value	95% CI for AOR
Gender			
Female	1		
Male	1.69	0.02	(1.09, 2.63)
Age			
15–24	1		
25–34	0.41	0.01	(0.22, 0.79)
35–44	0.32	0.00	(0.17, 0.6)
45–54	0.34	0.00	(0.17, 0.7)
55–64	0.24	0.01	(0.09, 0.66)
Drank alcohol			
Not reported drinking	1		
Yes reported drinking	2.07	0.00	(1.38, 3.09)
Constant	0.47	0.02	(0.25, 0.9)
Random intercept (region)			
Var(Uoi)	0.21		(0.04, 1.14)
Region ICC	0.06		(0.012, 0.257)

Among PLHIV aged 15 to 64 years, being male was 1.69 times more likely to present late for HIV diagnosis as compared to those female counterparts within the regions (AOR: 1.69; 95% CI: 1.09, 2.63). PLHIV in the age group of 25–34 years, 35–44 years, 45–54 years, and those 55–64 years had 59% (AOR: 0.41; 95% CI: 0.22, 0.79), 68.0% (AOR: 0.32; 95% CI: 0.17, 0.60), 66.0% (AOR: 0.34; 95% CI: 0.17, 0.70), and 76.0% (AOR: 0.24; 95% CI: 0.09, 0.66), respectively, lower odds of being lately diagnosed for HIV compared to those 15–24 years of age with in the regions (Table 5).

The odds of late diagnosis for HIV among PLHIV aged 15 to 64 who ever had used alcohol (AOR 2.07; 95% CI; 1.38, 3.09) was 2.07 times higher compared to those who had drink with in the regions.

### Disease stages among newly diagnosed

**Fig. 3:**
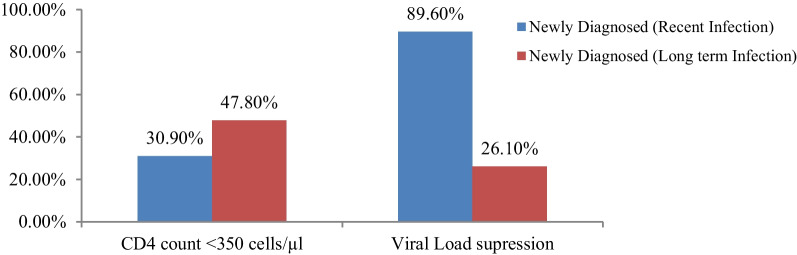
Stages of HIV disease among PLHIV who were newly diagnosed recent and long-term infection

Proportion of advanced immune suppression (CD4 count < 350 cells/µl) among newly diagnosed long-term PLHIV were significantly higher compared to those who were newly infected which accounted 47.8% and 30.9% (*P* = 0.001), respectively. Moreover, the median CD4 count among those who were newly diagnosed long-term PLHIV were lower 352 cells/mm^3^ compared to those who newly diagnosed new infections 457 cells/ mm^3^. Viral load suppression were significantly lower among those who were newly diagnosed long-term PLHIV (26.1%) compared to those newly diagnosed new infections (89.6%) (*P* = 0.001) (Fig. 3).

## Discussion

As the gateway to a continuum of HIV/AIDS services, timely HIV testing plays a central role in the fight against the HIV epidemic [16]. A recent study demonstrated the efficacy of early testing in immediately keeping patients on ART for the prevention of HIV transmission; and studies based on mathematical models suggest that UTT, consisting of treating every HIV-infected person as soon as diagnosis is made, can curb the epidemic. For all these reasons there is international consensus to expand HIV testing in resource limited countries like Ethiopia, as part of which generating evidence in this field will be crucial.

In this analysis, 29.4% of adults in urban Ethiopia were never tested for HIV. It was relatively higher compared to a study conducted in Malawi that revealed 25% of population reported having never been tested for HIV [17]. However, other similar studies reported higher HIV testing in Cóˆte d’Ivoire (50%) [16] and Mozambique (51%) [18]. This might be explained by program implementations among the different countries in a consistent time point.

Furthermore, our finding revealed that HIV testing differed across the administrative regions in Ethiopia. The proportion of adults who reported being never tested for HIV ranged from 18.8% in the Harari region to 20.3% in Afar to 32.6% in Oromia and to 69.1% in Somalia, which might be explained by the heterogeneity of HIV and program implementation among the different regional administrations in Ethiopia. This could also be explained by variations in the burden of HIV among the different regional administrations in the country which ranged from 0.1 to 5.6% [19]. This may highlight the importance of expanding testing among the high-burden regions including Gambella and Addis Ababa which are categorized under the hot spot areas and be included in Universal Testing in the country. The result of ICC of the two-level multilevel model revealed that about 6% of the variation in the likelihood of never tested for HIV was explained by the variation among the regions in urban Ethiopia. This calls for region-specific program interventions to enhanced HIV testing among high-burden priority regions.

Our study showed that the proportion of never tested among adult males was significantly higher than females. This is consistent with evidence from Zimbabwe, which showed a significant difference between male and female testing [20]. In contrast, a study conducted in Uganda reported a lower proportion of males (16%) than females was never tested [21]. Other studies have shown that women are more likely to report having ever been tested than men [16, 22, 23]. In our study, among adults aged 15 to 64 years being male was significantly associated with never tested for HIV compared with the female counterparts in the regions. This might be explained by the high rate of testing uptake for female as a result of PMTCT during antenatal follow-up [24]. Despite the high proportion of testing among women in urban Ethiopia, more than a quarter of women in reproductive age living in urban Ethiopia never tested. Women are among the priority population in Ethiopia and are parts of UT. Hence, this highlights the low testing of women in reproductive age in urban Ethiopia.

In the current study, the proportion of adults never tested was highest at age 15–24 years (47.8%) followed by 55–64 years (41.2%). Adults aged between 15–24, 35–44, 45–54 and 55–64 years were 2.49, 1.54, 3.14, and 5.26 times more likely to have never tested for HIV compared to 25–34 years, respectively. HIV incidence in the age 15–24 years is the highest among all age group (0.12) and HIV prevalence among the elderly accounts for 4.4%, compared to the prevalence in the general population of 0.96%. This might be explained by age-related behavioral factors for HIV testing [25, 26]. In this study, the low rate of testing among widowed could be worrisome for the program since the prevalence of HIV among the widowed is significantly higher 14.0% compared to 0.96% in the general and 3.0% in urban Ethiopia [27]. Widowed were also a priority population in the country.

Overall, 25.9% (95% CI: 21.7, 30.2) of the adults were diagnosed late for HIV in urban Ethiopia. This was high compared to the finding of a study conducted in United Kingdom (UK), which showed that 15.4% were diagnosed late [28], while our study is consistent with a report from Spain, which showed 30.4% were late presenters [12]. This might be explained by the different behavioral and environmental factors contributing to the different time to diagnosis [29].

Proportion of adults who tested late for HIV ranged from 38.1% in the Gambella region to 34.7% in Addis Ababa to 5.8% in Benishangul Gumuz and to 0.0% in Somalia, given the heterogeneity of HIV epidemic prevalence by region in Ethiopia, which ranged from 5.6% in Gambella followed by 3.3% in Addis Ababa and is less than 1% in Somali [30] and that the highest proportion of people with late diagnosis is in the high-burden regions, the program may consider focusing on devising strategies to increase testing in the high-burden regions.

In our study, the proportion of late diagnosis for HIV among adult males was significantly higher (36.8%; 95%: 28.0, 46.6) than females (20.8%; 95%: 17.3, 24.9). This result was not in line with a study in Mozambique which reported late diagnosis among males Vs females of 43.7% and 55.3% [31], respectively. However, our finding concurs with the findings of a study from South Africa which revealed that 57% of males and 43% of females were diagnosed late [32], showing that the proportion late diagnosis is higher among males.

The highest proportion of late diagnosis for HIV among adults living with HIV was in younger age 15–24 years (43.5%), followed by 25–34 years (26.6%) and in 45–54 years (25.2%), the lowest was in 55–64 years (19.5%). This may indicate the need for a more rigorous programmatic intervention among the adolescent and early diagnosis. Among PLHIV aged 15 to 64 years, males were 1.69 times more likely to present late for HIV diagnosis as compared to their female counterparts in the regions. Likewise, PLHIV in the age group of 25–34 years, 35–44 years, 45–54 years and those 55–64 years had 59.0%, 68.0%, 66.0% and 76.0%, respectively, and had lower odds of being diagnosed late for HIV compared to those 15–24 years of age. The different studies highlighted variations among the different age group, a study conducted in Durban revealed the high rate of late diagnosis at early adult stage and elderly which was consistent with this study [32]. Other study conducted in Uganda revealed late diagnosis among elderly [21]. This difference might be explained by the variations in behavioral factors among people in this age group [18].

In this study, the proportion of advanced immune suppression (CD4 count < 350 cells/µl) among newly diagnosed long-term infection PLHIV were significantly higher compared to those who were new infections which accounted 47.8% (95%CI = 33.2–52.1) and 30.9% (95%CI = 21.3–42.2), respectively. This was consistent with other studies which has been previously conducted [22, 33, 34]. This could impact on poor treatment outcome and could lead to opportunistic infections [10, 35]. A study conducted in south Africa also indicated there is 3.46 times risk of mortality among patients who were initiated for HAART with baseline CD4 count < 350 cells/mm^3^ [36].

Viral load suppression were significantly lower among those who were late diagnosed 26.1% (95%CI = 13.6–33.8) compared to those who were recently infected 89.6% (95%CI = 76.2, 93.4) which was also similar to other studies conducted in South Africa and Zimbabwe [35–37]. This may highlight the importance of early diagnosis for better therapeutic outcome of PLHIV during clinical management and also for the program prevention and control strategy [32, 38, 39]. The significantly low level of viral load suppression among newly diagnosed long-term infections could fuel the risk of community transmission (42).

## Strength and limitation of the study

One of the strengths of this study is, it provides evidence for a global priority public health issue in the context of Ethiopia using a large number of study participants (n = 18,926) across the different regions of urban Ethiopia using a cross-sectional household-based data collection. It also differentiates recent HIV infections from long-term HIV infections using different laboratory biomarkers and test algorithms.

However, this study did not consider all priority and at risk groups of HIV and further study may be required to specific group of population including female sex workers and drug users. Moreover, there has to be a continuous interpretation of this finding for the current use since data were collected by 2017–2018. Moreover, it has to be noted, the study is limited to urban setting in Ethiopia.

## Conclusion

With the aim of universal testing for high risk and priority population, the low HIV testing among widowed, elderly, young adolescent and women in urban Ethiopia calls for enhanced testing strategy among the high risk and priority population. Moreover, the low testing and high late diagnosis in Gambella and Addis Ababa regions which are high-burden priority regions may call for region-specific intervention. The high proportion of new diagnosis with long-term infection which led to advanced disease stages may impact on fueling community transmission and hinder treatment outcome among PLHIV.

## Data Availability

Data of this analysis are publicly available at https://phia.icap.columbia.edu/countries/ethiopia/.

## Abbreviations

AOR: Adjusted odds ratio
ARV: Antiretroviral
CI: Confidence interval
COR: Crude odds ratio
CSA: Central Statistics Agency
DBS: Dried blood spot
EPHI: Ethiopian Public Health Institute
HIV: Human immunodeficiency virus
HTS: HIV testing service
ICC: Intra-cluster correlation
OD: Optical density
PLHIV: People living with HIV
PMTCT: Prevention of mother to child HIV transmission
SERO: Scientific and Ethical Review Office
UT: Universal testing
UTT: Universal test and treat
VL: Viral load
VLS: Viral load suppression

## Operational definition

Adults: For the purposes of this study, adults are defined as the population aged 15-64 years.
Antiretroviral (ARV): A type of medication used in the treatment of HIV.
Antiretroviral therapy (ART): Treatment with antiretroviral (ARV) drugs that inhibit the ability of HIV to multiply in the body, leading to improved health and survival among HIV-positive persons.
Enumeration area (EA): A limited geographic area defined by the national statistical authority and the primary sampling unit for the Population-Based HIV Impact Assessment (PHIA) surveys.
HIV viral load (VL): The concentration of HIV virus particles in the blood, expressed as copies per milliliter (copies/mL).
HIV viral load suppression (VLS): An HIV VL of less than 1000 copies per mL
Risk group for universal testing: By gender (female); by age (Elderly (>50 years old) and young, by marital status (Widowed or separated), by geography (high risk and burden regions; Gambella and Addis Ababa).
Late diagnosis and new infection: PLHIV who were classified by LAg avidity assay
